# Spice up the hypertension diet - curcumin and piperine prevent remodeling of aorta in experimental L-NAME induced hypertension

**DOI:** 10.1186/1743-7075-8-72

**Published:** 2011-10-17

**Authors:** Livia Hlavačková, Andrea Janegová, Olga Uličná, Pavol Janega, Andrea Černá, Pavel Babál

**Affiliations:** 1 Department of Pathology, Faculty of Medicine Comenius University, Sasinkova 4, 81372 Bratislava, Slovakia; 2 Pharmacobiochemical laboratory of 3rd Department of Internal Medicine, Faculty of Medicine Comenius University, Sasinkova 4, 81372 Bratislava, Slovakia; 3 Institute of Normal and Pathological Physiology, Slovak Academy of Sciences, Sienkiewiczova 1, 81101 Bratislava, Slovakia

**Keywords:** piperine, curcumin, L-NAME, hypertension, aorta, remodeling

## Abstract

**Background:**

Increase of blood pressure is accompanied by functional and morphological changes in the vascular wall. The presented study explored the effects of curcuma and black pepper compounds on increased blood pressure and remodeling of aorta in the rat model of experimental NO-deficient hypertension.

**Methods:**

Wistar rats were administered for 6 weeks clear water or L-NAME (40 mg/kg/day) dissolved in water, piperine (20 mg/kg/day), curcumin (100 mg/kg/day) or their combination in corn oil by oral gavage. The systolic blood pressure was measured weekly. Histological slices of thoracic aorta were stained with hematoxylin and eosin, Mallory's phosphotungstic acid hematoxylin (PTAH), orcein, picrosirius red and van Gieson staining and with antibodies against smooth muscle cells actin. Microscopic pictures were digitally processed and morphometrically evaluated.

**Results:**

The increase of blood pressure caused by L-NAME was partially prevented by piperine and curcumin, but the effect of their combination was less significant. Animals with hypertension had increased wall thickness and cross-sectional area of the aorta, accompanied by relative increase of PTAH positive myofibrils and decrease of elastin, collagen and actin content. Piperine was able to decrease the content of myofibrils and slightly increase actin, while curcumin also prevented elastin decrease. The combination of spices had similar effects on aortic morphology as curcumin itself.

**Conclusions:**

Administration of piperine or curcumin, less their combination, is able to partially prevent the increase of blood pressure caused by chronic L-NAME administration. The spices modify the remodeling of the wall of the aorta induced by hypertension. Our results show that independent administration of curcumin is more effective in preventing negative changes in blood vessel morphology accompanying hypertensive disease.

## Background

N-nitro-L-arginine-methylester (L-NAME) is a nonspecific inhibitor of all three NO synthase (NOS) isoforms (neuronal - nNOS; inducible - iNOS; endothelial - eNOS) and causes an increase of blood pressure in a dose-dependent manner when administered to the experimental animals [1,2]. The adaptation process to the increased blood pressure may include several morphological changes in the blood vessel wall. Hypertension causes thickening of the vascular wall [3,4], however, the role of blood pressure in the inner diameter enlargement is currently unclear since some claim its importance [5,6] while others present its insignificant role in this process [7,8].

Most of the morphological changes appear in the *tunica media *formed by smooth muscle cells (SMC) and the extracellular matrix (ECM). Under normal conditions mature SMC exhibit low synthetic activity [9] but unlike skeletal and heart muscle, the vascular SMCs are able to change their phenotype and start producing large amounts of ECM components and increase their proliferation and migration [10]. Actin polymerization is one of the important SMC phenotype changes [11].

Collagens in the aortic wall are represented mainly by collagens of type I and type III [12]. Collagen type I (Col I) is mechanically firm and hypertension induced by long term administration of L-NAME leads to an increase of its synthesis in blood vessels [13]. Collagen type III (Col III) forms a net of fine fibers which enables to maintain blood vessel elasticity and their damage results in increased vessel wall rigidity [14,15]. The protein responsible for the blood vessel elasticity is elastin [16] which is in comparison to collagen an extremely stabile polymer with low level biological turn-over [17]. When mechanically damaged by hypertension it can be newly synthesized in higher amounts [18].

Nutrition and lifestyle play a vital role in prevention of hypertension and therefore there is a continuous search for dietary components having positive effect on blood pressure. Curcumin is the main polyphenol of *Curcuma longa*, a spice of yellow color. It is currently known due to its chemopreventive [19], antioxidant and antiinflammatory [20] properties. Moreover, curcumin is able to inhibit proliferative activity of vascular SMC *in vitro *and induce their apoptosis and thus to participate in prevention of pathological changes in blood vessels [21].

Piperine is responsible for the pungency of spices gained from *Piper nigrum *and *Piper longum *(sources of black or long pepper) [22]. Its main biological significance lies in its ability to influence metabolism of other substances (including drugs) and if consumed together it can significantly increase the bioavailibility of curcumin [23]. The hypotensive effect of piperine on blood pressure has been verified after acute intravenous administration [24] or per orally in high doses (50 mg/kg) [25]. However, piperine in higher concentrations leads to increased hydroxyl radical production while in low concentrations it acts as an antioxidant [26] and it is important to establish whether piperine is able to influence the blood pressure after peroral administration of lower doses.

The aim of the presented study is to evaluate the preventive effects of piperine and curcumin on blood pressure increase and the pathological changes in blood vessel morphology in the experimental hypertension induced by L-NAME administration.

## Methods

### Experimental protocol

All procedures and experimental protocols were approved by an ethical committee and conform to the European Convention on Animal Protection and Guidelines on Research Animal Use. The animals were housed in an air-conditioned room at a steady temperature (22-24°C) and humidity (45-60%) on a 12:12 h light/dark cycle and maintained on a standard pellet diet and tap water *ad libitum*. During the experiment, three animals (one from L-NAME group, two from L-NAME + curcumin + piperine group) died from technical problems.

Adult 12-week-old male Wistar rats were divided into 8 groups with 6 animals in each group: control group (Con, receiving pure water and corn oil), group treated with 40 mg/kg/day of L-NAME (L) dissolved in the drinking water and administered orally; groups receiving piperine (20 mg/kg/day) in corn oil by oral gavage with L-NAME (LP) or without it (P); groups receiving curcumin (100 mg/kg/day) in corn oil (max 0.4 ml per animal) by oral gavage with L-NAME (LC) or without it (C); groups receiving curcumin and piperine in the same manner with L-NAME (LCP) or without it (CP). The dose of curcumin was chosen according to previous positive results on abdominal aorta [27]. The selected dose of piperine was reported to be able to enhance the bioavailibility of curcumin [28].

All compounds were administered daily for 6 weeks and the experiment was ended by an overdose of thiopental. Daily water consumption and the body weight were estimated one week before the experiment and controlled during the treatment. There were no statistically significant changes of the body weights of animals in different experimental groups at the beginning or at the end of the experiment.

### Blood Pressure Measurement

The systolic blood pressure (BP) was measured non-invasively in awaked animals by the tail-cuff plethysmography by using blood pressure module (NIBP Controller, ADInstruments, Spechbach, Germany) connected through the manometer and Powerlab 8/30 module (ADInstruments, Spechbach, Germany) to the computer. The last measurement was taken at the end of the 5th week of administration. The final value was calculated from five successive measurements.

### Histology Staining

Specimens of the middle part of thoracic aorta were fixed 24 h in 10% formalin, routinely processed in paraffin and 5 μm thick slices were stained with hematoxylin and eosin, Mallory's phosphotungstic acid hematoxylin (PTAH), van Gieson collagen staining and orcein elastin staining using standard procedures.

Smooth muscle actin was detected by mouse monoclonal antibodies (DAKO) diluted 1:100 in Antibody Dilutent (DAKO) applied on specimens with previously heat-retrieved epitopes in 10 mmol/L Tris buffer, 1 mmol/L EDTA, pH 9.0. The slides were incubated with the primary antibody for one hour at laboratory temperature, then washed in TBS (pH 7.4) and incubated with anti-mouse peroxidase complex (EnVision^® ^kit, DAKO), washed in TBS and the color reaction was developed with diaminobenzidine (DAKO). The immunohistochemical staining was performed in DAKO Autostainer staining automat.

Sirius red F3BA dissolved in a saturated picric acid stains collagens type I and III, which can be distinguished with the use of polarized light, and their content evaluated by computer assisted morphometry is in concordance with the results obtained by immunohistochemistry and evaluation of mRNA levels [29,30]. Modified technique with picrosirius red [31] was used as follows: the slides were submerged in 0.2% phosphomolybdic acid, then stained with 0.1% sirius red F3BA in a saturated water solution of picric acid for 90 min, washed in 0.01 N HCl, dehydrated and mounted. Slides were then viewed under polarized light, in which Col I has red to yellow color because of thickness of its bundles, while thin collagen type III. fibers are green [30].

If not stated otherwise, all chemicals were purchased in Sigma Chemie (Germany).

### Digital Morphometry

All slides were evaluated in a Leica light microscope (Leica Systeme, Wetzlar, Germany). The slides stained by hematoxilin and eosin were photographed by digital photographic camera (S 50, Canon, Japan) using the same conditions (microscope magnification 45× and camera settings) for each slide and for Burker cell counting chamber. Based on the known size of the Burker chamber's grid the evaluating programme (ImageJ 1.42q, National Institute of Health, USA) was pre-set to calculate the results in SI units. Internal circumference along the vessel *lamina elastic interna *and external circumference along *lamina elastica externa *were then measured. Inner diameter, tunica media thickness and its cross-sectional area (CSA) were calculated.

For all other stainings, at least five random places were selected on each slide and documented. The pictures were digitally processed in Active pixels software (Idea systems, USA) to limit the measurement to aortic media (the background and other parts of aorta were deleted) and afterwards evaluated with the ImageJ software. Threshold values were determined for the colors or the intensity of staining representing the analyzed compounds of aortic media. Cross-sectional area of media was estimated for every taken picture and percentage of the specific color covering that particular area was measured. Proportional amounts were then matched together with results obtained by the morphometric evaluation (CSA size in square millimeters) used in order to calculate the "absolute" amount of the stained components of aortic media, as well as the amount of unstained components.

### Statistics

The results were statistically analyzed by GraphPad Prism (GraphPad Software, USA) using two-way ANOVA with Bonferroni test for blood pressure (time and given substances being the two variables) or one-way ANOVA with Keuls-Neumann test, and expressed as mean ± standard deviation. Values with p < 0.05 were considered significant.

## Results

### Blood pressure

**Figure 1:**
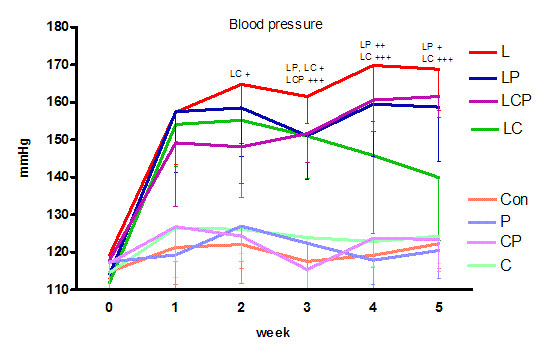
**Effect of piperine and curcumin on systolic blood pressure in L-NAME-induced hypertension**. Con - control, L - L-NAME induced hypertension, P - piperine, C - curcumin. + p < 0.05, ++ p < 0.01, +++ p < 0.001 versus L.

At the beginning of the experiment (week 0) the systolic blood pressure was without any differences among the groups. Administration of curcumin, piperine and their combination caused no significant changes in blood pressure. Administration of L-NAME induced a significant increase in blood pressure already after the first week (to 157.4 ± 14.0 mm Hg) and continuing administration caused even further increase (168.8 ± 11.1 mm Hg the 5th week) (Figure 1).

Treatment with piperine caused a significant decrease in blood pressure in comparison to the L-NAME group following the third week of administration, however, the blood pressure remained above the control until the end of the experiment. Curcumin treatment caused a significant drop of the increased blood pressure starting the second week and this reduction continued in the following weeks reaching a decrease of 28.9 mm Hg at the end of the experiment. The combination of spices showed a tendency to decrease the blood pressure but these changes were statistically significant only in the third week (Figure 1).

### Morphometry

**Figure 2:**
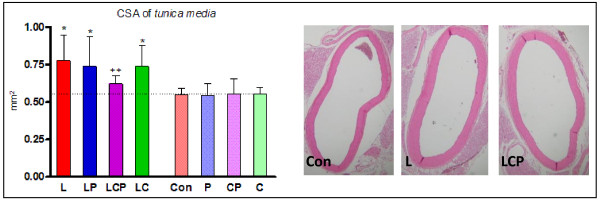
**Changes in cross sectional area (CSA) of the media of aorta induced by hypertension and the effect of piperine and curcumin in the graphic form (left) and examples of the aorta stained with hematoxylin and eosin (right)**. Con - control, L - L-NAME induced hypertension, P - piperine, C - curcumin. * p < 0.05 versus Con, ++ p < 0.01 versus L.

Administration of the spices alone did not cause any significant change in the inner aortic diameter, aortic media thickness or the cross section area (CSA) when compared to the control. Six weeks of L-NAME-induced hypertension caused a significant enlargement of the aorta and co-administration of either curcumin or piperine was not able to prevent it. Combination of the spices was able to significantly reduce the aortic wall thickness in animals with hypertension to average values of the CSA that did not significantly differ from the control (Figure 2).

### Fibrilar proteins stained by Mallory's phosphotungstic acid hematoxylin (PTAH)

**Figure 3:**
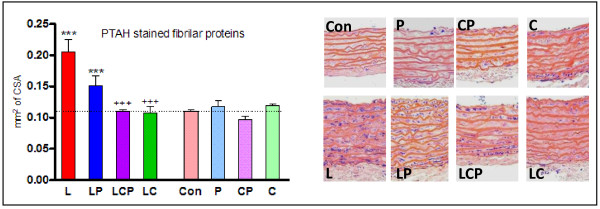
**The absolute amount of myofibrils in the aortic media identified with phosphotungstic acid hematoxylin was significantly increased in hypertensive animals (L), curcumin caused their return to the level of controls**. Graphic form of the results (left) obtained from aorta specimens stained with PTAH (right). CSA - cross sectional area, Con - control, L - L-NAME induced hypertension, P - piperine, C - curcumin. +++ p < 0.001 versus L, . *** p < 0.001 versus Con.

Neither of the spices alone caused any significant changes in the proportional amount of myofibrils stained by PTAH in the aortic media. L-NAME-induced hypertension inflicted a significant gain in their relative content which was reduced by simultaneous administration of curcumin and/or piperine, with curcumin being significantly more effective than piperine (Table 1). The absolute amount of PTAH-positive myofibrils in the CSA was not affected by the spices alone but was increased in hypertensive animals. Piperine had no effect on this increase, curcumin and combination of the spices brought about a significant decrease of the absolute amount of PTAH positive myofibrils to the control level (Figure 3).

**Table 1:** Content of various proteins detected in the aortic media

	L	LP	LCP	LC	Con	P	CP	C
**PTAH**	26.38 ± 11.65*	21.06 ± 10.52 +	17.69 ± 5.02 ++	15.01 ± 8.52 +++	20.07 ± 7.12	21.58 ± 13.41 +	17.45 ± 4.88 ++	21.50 ± 6.92 +

**actin**	18.87 ± 13.19***	24.81 ± 10.39*. +	26.81 ± 8.41 ++	27.76 ± 8.50 +++	31.03 ± 9.00	32.69 ± 7.34 +++	30.48 ± 8.11 +++	27.96 ± 6.25 ++

**elastin**	25.75 ± 3.93***	28.06 ± 3.92***	33.73 ± 3.09 +++	30.95 ± 8.36 +	34.12 ± 7.88	33.26 ± 9.02 +++	33.78 ± 9.40 +++	34.74 ± 5.57 +++

**collagen I**	12.48 ± 6.77**	10.60 ± 8.23***	10.85 ± 3.45***	7.22 ± 4.76***	20.20 ± 12.10	22.67 ± 7.18 ++	18.67 ± 11.09 +	22.11 ± 10.12 ++

**collagen III**	0.23 ± 0.16*	0.20 ± 0.17**	0.30 ± 0.18*	0.22 ± 0.24**	0.50 ± 0.39	0.61 ± 0.44 +	0.60 ± 0.51 ++	0.58 ± 0.43 ++

**total collagen**	12.75 ± 6.89**	10.81 ± 8.36***	11.15 ± 3.56***	7.44 ± 4.95***	20.71 ± 12.27	23.29 ± 7.41 +++	19.27 ± 11.46 +++	22.69 ± 10.38 +++

**van Gieson**	15.14 ± 6.78***	17.45 ± 4.22***	17.35 ± 2.08***	13.98 ± 4.15***	25.57 ± 6.27	27.01 ± 5.42 +++	25.20 ± 3.34 +++	26.08 ± 3.82 +++

**CSA**	0.77 ± 0.17*	0.73 ± 0.19*	0.62 ± 0.05 ++	0.73 ± 0.13*	0.55 ± 0.04	0.54 ± 0.07 +	0.55 ± 0.09 +	0.55 ± 0.03 +

**thickness**	0.14 ± 0.03*	0.14 ± 0.02**	0.12 ± 0.01	0.14 ± 0.02***	0.11 ± 0.01	0.10 ± 0.01 ++	0.11 ± 0.01 +	0.11 ± 0.01 +

**inner diameter**	1.42 ± 0.09	1.51 ± 0.15	1.43 ± 0.03	1.47 ± 0.12	1.54 ± 0.09	1.53 ± 0.12	1.58 ± 0.11	1.49 ± 0.13

The table summarizes the values of the relative content of various proteins in the media of the aorta expressed in% of the total cross sectional area (CSA), which is given in mm^2^, thickness and inner diameter in mm. Con - control, L - L-NAME induced hypertension, P - piperine, C - curcumin. + p < 0.05, ++ p < 0.01, +++ p < 0.001 versus L; * p < 0.05 versus Con, ** p < 0.01 versus Con, *** p < 0.001 versus Con.

### Smooth muscle cell actin

**Figure 4:**
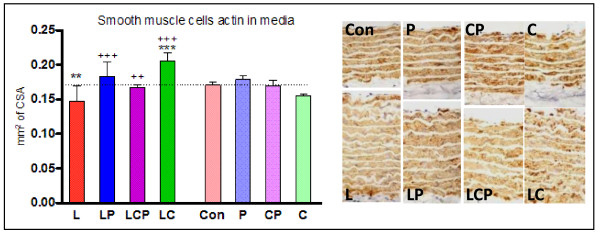
**Smooth muscle cell actin in the media of aorta was decreased in hypertensive animals, both spices substances reversed this decrease**. Immunohistochemical detection of smooth muscle actin (right) and evaluation in graphic form (left). CSA - cross sectional area, Con - control, L - L-NAME induced hypertension, P - piperine, C - curcumin. ++ p < 0.01 versus L,+++ p < 0.001 versus L, ** p < 0.01 versus Con, *** p < 0.001 versus Con.

The proportional content of smooth muscle cell actin in the aortic media was not changed by curcumin and/or piperine administration. Six weeks of L-NAME-induced hypertension lead to a significant decrease in the actin relative amount. Piperine administration had no significant effect but the relative content of actin was normalized by curcumin (with or without piperine) to the level of controls (Table 1). The absolute content of actin counted in square millimeters of aortic media cross section area was not influenced by the spices alone and was decreased in hypertensive animals. Piperine with or without curcumin returned the absolute amount of actin in these animals to the control level while curcumin led to an increase of the actin amount significantly above the level of controls (Figure 4).

### Collagens stained by van Gieson and picrosirius red staining

**Figure 5:**
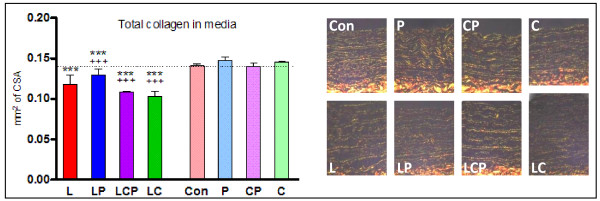
**The total content of collagen in the aorta was significantly decreased in hypertensive animals**. The spices did not substantially correct this drop. Graph (left) with results from van Gieson staining, specimens stained with picrosirius red evaluated in polarized light (right). Con - control, L - L-NAME induced hypertension, P - piperine, C - curcumin. ** p < 0.01 versus Con, *** p < 0.001 versus Con.

In specimens stained with picrosirius red staining the spices alone did not change the proportion of collagen in comparison to the control. L-NAME-induced hypertension led to a significant decrease in the relative content of total collagen and also of Col I and Col III, in the aortic media. These changes were not prevented by the spices co-administration (Table 1). The staining by van Gieson provided data similar to those obtained with picrosirius red. The absolute amount of collagen was not changed by the spices and was decreased in hypertensive animals. Piperine administration induced a significant decrease of the absolute amount of collagen in these animals. Curcumin administration (with or without piperine) caused a further decreased of the absolute amount of collagen in the aortas of hypertensive animals (Figure 5).

### Elastin stained by orcein

**Figure 6:**
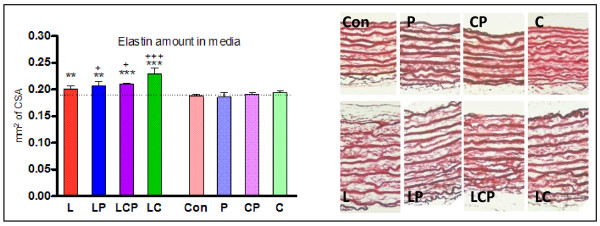
**Content of elastin in the aortic media was significantly increased in animals with hypertension, the spices induced its further raise**. Graphic form of the results (left) obtained from aorta specimens stained with orcein (right). CSA - cross sectional area, Con - control, L - L-NAME induced hypertension, P - piperine, C - curcumin. + p < 0.05 versus L,+++ p < 0.001 versus L, ** p < 0.01 versus Con, *** p < 0.001 versus Con.

Administration of the spices did not change the proportional amount of elastin in the aortic media. This was significantly decreased in hypertensive animals. The effect of L-NAME-induced hypertension was not prevented by piperine alone, but curcumin with or without piperine caused an increase of elastin proportional content reaching the level of controls when combined with piperine (Table 1). The calculated absolute amount of elastin disclosed that spices alone caused no change in its amount. Surprisingly, the absolute content of elastin was increased by L-NAME-induced hypertension and administration of curcumin and/or piperine lead even to its further increase (Figure 6).

### Ground substance

Mathematically calculated changes of the ground substance were expressed in percentage and in absolute values (squared millimeters) of CSA of the aortic media. Since only average numbers were used for the calculation no statistical significance was stated and the results are considered as illustrational.

**Figure 7:**
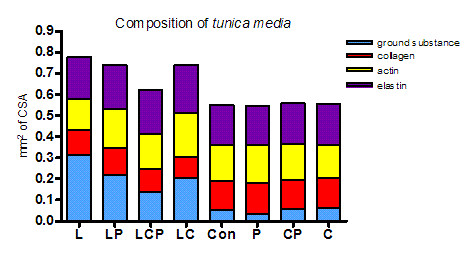
**The amount of the ground substance of the aorta was increased in animals with L-NAME-induced hypertension and this increase was reduced by both piperine and by curcumin, more effectively in their combination**. Con - control, L - L-NAME induced hypertension, P - piperine, C - curcumin.

After administration of the spices the relative contents of the components of the aortic media as well as of the ground substance were not changed when compared to the control. Long term L-NAME administration with hypertension development caused an increase of the proportional amount of the ground substance (from 9.28% to 40.24%) as well as its absolute content (from 0.05 mm^2 ^to 0.31 mm^2^). The absolute amount of the ground substance in the aortic media of animals with induced hypertension changed after six weeks of spices administration as follows: piperine 29.68% (0.21 mm^2^), curcumin 27.31% (0.20 mm^2^) and combination of the spices 22.11% (0.13 mm^2^) (Figure 7).

## Discussion

Six weeks of L-NAME administration causes a chronic increase in blood pressure in rats previously described by other authors even after shorter duration of the experiment [32]. The increase in blood pressure after L-NAME administration is connected with inhibition of NO synthases and decrease in NO production [33,34]. Down regulation of eNOS may also participate at lower NO availability, however, the changes in iNOS expression may vary depending on the length of L-NAME administration [35,24].

Administration of piperine or curcumin to animals with L-NAME-induced hypertension lead to a decrease of blood pressure, curcumin causing a more significant drop. Similar hypertension reducing effect of curcumin has been reported recently by Nakmreong et al. [34]. Another study [25] registered a significant decrease of blood pressure after intravenous piperine administration. Piperine treatment was effective also in lowering blood pressure in rats with L-NAME-induced experimental hypertension described by Kumar et al. [36]. The combination of curcumin and piperine in our experiment did not cause a statistically significant blood pressure decrease (with the exception of the third week) but it is apparent that it copies the course of the piperine graph curve. These differences in the results are probably related to different mechanisms of effect by the spices on blood pressure.

According to Kumar et al. [36] piperine is able to increase the NOx production, although it is unclear which NO synthase is responsible for this effect. Taqvi et al. [25] reported that the effect of piperine on blood vessels is similar to that of calcium channel blocker verapamil. By this mechanism piperine probably prevents the curcumin to manifest its full effect through eNOS, the expression of which is increased by curcumin [34]. Inducible NOS remains during the L-NAME administration an important source of hemodynamically active nitric oxide and apparently it is not able to prevent the hypertension or the pathological vascular reactivity [37]. While curcumin decreases iNOS expression [38-40], stimulation of eNOS expression by this spices is likely to be the main factor in reducing the increased blood pressure induced by L-NAME administration [34,41]. NO-deficient hypertension in our experiment is accompanied by morphological changes of the blood vessel wall. According to our results and the results of others [7,8,41,42], L-NAME administration does not affect the inner diameter of the thoracic aorta but it increases the cross-sectional area and the thickness of the aortic media. This change appears mostly due to the increase of ground substance and water [43,44] that can affect vessel wall elasticity and the changes of the proportional amount of other components in the media of the aorta. Described remodeling effects of L-NAME-induced hypertension could be effectively prevented only by the combination of piperine and curcumin while the spices given independently did not have a significant effect. Reduction of oxidative stress in the aortic media by the spices [34,23] might contribute to reduction of water content in the ground substance and leading to reduction of the crossectional area of the aorta in hypertensive animals. Inhibitory effect of piperine on vascular remodeling has been described through activation of superoxide dismutase [45] or differential expression of calcium regulating channels [46]. Both spices influence remodeling by different mechanisms, thus, their combination resulted in an additive reducing effect on vascular remodeling.

The relative content of elastin and collagen was decreased in the aortas of hypertensive animals. The decrease of relative elastin content might be the result of the effect of increased blood pressure and has been also detected in spontaneously hypertensive rats [47]. The absolute amount of elastin in the aortic media, however, was increased when compared to control, which could be ascribed to the increased synthetic activity of vascular wall cells as a response to the increased fatigue of elastin [48] depicted also in other models of experimental hypertension [18]. The spices, and especially curcumin alone or in combination with piperine, caused a further increase of elastin content in the blood vessel wall probably also due to the inhibitory effect on elastases [49,50] or on the matrix metalloproteinase 2 [51]. In case of curcumin the just mentioned effects probably lead also to the significant increase of the relative amount of elastin in our experimental setting.

The changes of collagen content in the aorta will relate to the used experimental model, as some authors described its increase [52] while others did not observe any changes [48,53]. Decrease of collagen content seen in our experimental setting was probably caused by increased activity of matrix metalloproteinases, the higher activity of which has been described in spontaneously hypertensive rats [54]. The effect of the spices on the degrading enzymes as well as on the synthetic activity reflected the absolute amount of collagen in the aorta. Piperine in this process presumably inhibits the matrix metalloproteinase 13 and thus may support the collagen amount increase [55]. However, this effect is not sufficient to compensate the probable inhibition of collagen synthesis by curcumin [56].

The spices elicited a significant effect on smooth muscle cells. L-NAME-induced hypertension caused a decrease in the amount of actin. This might be the result of increased proliferation of SMCs synthesizing less of this protein [57]. At the same time the proportional amount of myofibrils increased in muscle cells as we already have described in the NO-deficient hypertension [32], probably because of the changes in globular and fibrilar actin [58]. This conclusion was drawn from the differences resulting from the used detection methods. The antibodies bind to actin regardless its quarterly structure [59], which on the other hand is crucial for the phosphotungstic acid hematoxylin staining [60]. Curcumin has been shown to suppress the vascular smooth muscle cells migration and proliferation [61] and to differentially interfere with proteins translation [62], which may correlate with correction of the fibrilar proteins content in the aortic media.

The effect of hypertension on myofibrils was partially preventable by curcumin and/or piperine administration, piperine being less effective than curcumin. The effect of both spices can be explained by influencing the actin polymerization [21]. Piperine is able to block the influx of calcium which is needed for the change of G-actin to F-actin [63]. The administration of curcumin leads to an increase of actin content above the level of controls and it can be assumed that this effect is caused by an increased reaction of the injured muscle cells to stimulation of its synthesis. When the spices are combined, the protective effect of piperine on muscle cells [57] probably prevents the overreaction of SMSs to curcumin and leads to the normalization of the actin amount in the vessel wall.

In conclusion, the increase of blood pressure caused by L-NAME can be partially prevented by piperine or curcumin, the result of their combination being less significant. L-NAME-induced hypertension leads to an increase of the aortic media thickness and its cross-sectional area, which are accompanied by the increased proportional amount of PTAH-positive myofibrils, decrease of elastin, collagen and actin. Piperine is able to decrease the content of myofibrils and slightly raise the proportion of actin, while curcumin also prevents the decrease of elastin. The combination of the spices has a similar effect on the vessel morphology as curcumin alone. The effects on the morphology of the aorta wall may be mediated by different mechanisms of action of the spices. Our results show that independent administration of curcumin is more effective in preventing negative changes in blood vessel morphology accompanying hypertensive disease.

## Competing interests

The authors declare that they have no competing interests.

## Authors' contributions

LH conceived of the study, and participated in its design and coordination and drafted the manuscript, AJ carried out the immunohistochemical staining and evaluation of the slides, UO was involved animal handling and obtaining of specimens, PJ carried out the experiment preparation, was involved in statistical analysis and slides evaluation, AČ was involved in animal handling, obtaining of specimens and slides evaluation, PB overviewed the experiment, was involved in slides evaluation, manuscript drafting and has given final approval of the manuscript to be published. All authors read and approved the final manuscript.

## References

[B1] Arnal JF el Amrani AI Chatellier G Ménard J Michel JB Cardiac weight in hypertension induced by nitric oxide synthase blockade Hypertension 1993 22 380 7 8349331 10.1161/01.hyp.22.3.380

[B2] Bernátová I Pechánová O Kristek F Mechanism of structural remodelling of the rat aorta during long-term NG-nitro-L-arginine methyl ester treatment Jpn J Pharmacol 1999 81 99 106 10.1254/jjp.81.99 10580377

[B3] Wolinsky H Response of the rat aortic media to hypertension. Morphological and chemical studies Circ Res 1970 26 507 22 5435712 10.1161/01.res.26.4.507

[B4] Xu C Lee S Singh TM Sho E Li X Sho M Masuda H Zarins CK Molecular mechanisms of aortic wall remodeling in response to hypertension J Vasc Surg 2001 33 570 8 10.1067/mva.2001.112231 11241129

[B5] MacSweeney ST Powell JT Greenhalgh RM Pathogenesis of abdominal aortic aneurysm Br J Surg 1994 81 935 41 10.1002/bjs.1800810704 7922083

[B6] Vardulaki KA Walker NM Day NE Duffy SW Ashton HA Scott RA Quantifying the risks of hypertension, age, sex and smoking in patients with abdominal aortic aneurysm Br J Surg 2000 87 195 200 10.1046/j.1365-2168.2000.01353.x 10671927

[B7] Mitchell GF Lacourcière Y Ouellet JP Izzo JL Jr Neutel J Kerwin LJ Block AJ Pfeffer MA Determinants of elevated pulse pressure in middle-aged and older subjects with uncomplicated systolic hypertension Circulation 2003 108 1592 1598 10.1161/01.CIR.0000093435.04334.1F 12975261

[B8] Agmon Y Khandheria BK Meissner I Schwartz GL Sicks JD Fought AJ O'Fallon WM Wiebers DO Tajik AJ Is aortic dilatation an atherosclerosis-related process? J Am Coll Cardiol 2003 42 1076 1083 10.1016/S0735-1097(03)00922-7 13678934

[B9] Owens GK Regulation of differentiation of vascular smooth muscle cells Physiol Rev 1995 75 487 517 7624392 10.1152/physrev.1995.75.3.487

[B10] Owens GK Kumar MS Wamhoff BR Molecular regulation of vascular smooth muscle cell differentiation in development and disease Physiol Rev 2004 84 767 801 10.1152/physrev.00041.2003 15269336

[B11] Doevendans PA van Eys G Smooth muscle cells on the move: the battle for actin Cardiovasc Res 2002 54 499 502 10.1016/S0008-6363(02)00395-4 12031695

[B12] Satta J Juvonen T Haukipuro K Juvonen M Kairaluoma MI Increased turnover of collagen in abdominal aortic aneurysms, demonstrated by measuring the concentration of the aminoterminal propeptide of type III procollagen in peripheral and aortal blood samples J Vasc Surg 1995 22 155 60 10.1016/S0741-5214(95)70110-9 7637115

[B13] Chatziantoniou C Boffa JJ Ardaillou R Dussaule JC Nitric oxide inhibition induces early activation of type I collagen gene in renal resistance vessels and glomeruli in transgenic mice. Role of endothelin J Clin Invest 1998 101 2780 9 10.1172/JCI2132 9637712 PMC508869

[B14] Sassi ML Carboxyterminal degradation product of type I collagen 2001 Oulu: Oulu university press

[B15] Silver FH Horvath I Foran DJ Viscoelasticity of the vessel wall: the role of collagen and elastic fibers Crit Rev Biomed Eng 2001 29 279 301 11730097 10.1615/critrevbiomedeng.v29.i3.10

[B16] Gosline J Lillie M Carrington E Guerette P Ortlepp C Savage K Elastic proteins: biological roles and mechanical properties Philos Trans R Soc Lond B Biol Sci 2002 357 121 32 10.1098/rstb.2001.1022 11911769 PMC1692928

[B17] Debelle L Tamburro AM Elastin molecular description and function Int J Biochem Cell Biol 1999 31 261 72 10.1016/S1357-2725(98)00098-3 10216959

[B18] Keeley FW Alatawi A Response of aortic elastin synthesis and accumulation to developing hypertension and the inhibitory effect of colchicine on this response Lab Invest 1991 64 499 507 2016856

[B19] Sarkar FH Li Y Using chemopreventive agents to enhance the efficacy of cancer therapy Cancer Res 2006 66 3347 50 10.1158/0008-5472.CAN-05-4526 16585150

[B20] Jovanovic SV Steenken S Boone CW Simic MG H-Atom transfer is a preferred antioxidant mechanism of curcumin J Am Chem Soc 1999 121 9677 9681 10.1021/ja991446m

[B21] Chen HW Huang HC Effect of curcumin on cell cycle progression and apoptosis in vascular smooth muscle cells Br J Pharmacol 1998 124 1029 40 10.1038/sj.bjp.0701914 9720770 PMC1565483

[B22] Srinivasan K Black pepper and its pungent principle-piperine: a review of diverse physiological effects Crit Rev Food Sci Nutr 2007 47 735 48 10.1080/10408390601062054 17987447

[B23] Suresh D Srinivasan K Tissue distribution & elimination of capsaicin, piperine & curcumin following oral intake in rats Indian J Med Res 2010 131 682 91 20516541

[B24] Luvarà G Pueyo ME Philippe M Mandet C Savoie F Henrion D Michel JB Chronic blockade of NO synthase activity induces a proinflammatory phenotype in the arterial wall: prevention by angiotensin II antagonism Arterioscler Thromb Vasc Biol 1998 18 1408 16 10.1161/01.ATV.18.9.1408 9743229

[B25] Taqvi SI Shah AJ Gilani AH Blood pressure lowering and vasomodulator effects of piperine J Cardiovasc Pharmacol 2008 52 452 8 10.1097/FJC.0b013e31818d07c0 19033825

[B26] Mittal R Gupta RL In vitro antioxidant activity of piperine Methods Find Exp Clin Pharmacol 2000 22 271 4 10.1358/mf.2000.22.5.796644 11031726

[B27] Parodi FE Mao D Ennis TL Pagano MB Thompson RW Oral administration of diferuloylmethane (curcumin) suppresses proinflammatory cytokines and destructive connective tissue remodeling in experimental abdominal aortic aneurysms Ann Vasc Surg 2006 20 360 8 10.1007/s10016-006-9054-7 16779518

[B28] Shoba G Joy D Joseph T Majeed M Rajendran R Srinivas PS Influence of piperine on the pharmacokinetics of curcumin in animals and human volunteers Planta Med 1998 64 353 6 10.1055/s-2006-957450 9619120

[B29] Pauschinger M Knopf D Petschauer S Doerner A Poller W Schwimmbeck PL Kühl U Schultheiss HP Dilated cardiomyopathy is associated with significant changes in collagen type I/III ratio Circulation 1999 99 2750 6 10351968 10.1161/01.cir.99.21.2750

[B30] Allon I Vered M Buchner A Dayan D Stromal differences in salivary gland tumors of a common histopathogenesis but with different biological behavior: a study with picrosirius red and polarizing microscopy Acta Histochem 2006 108 259 64 10.1016/j.acthis.2006.05.007 16899283

[B31] Dolber PC Spach MS Conventional and confocal fluorescence microscopy of collagen fibers in the heart J Histochem Cytochem 1993 41 465 9 10.1177/41.3.7679127 7679127

[B32] Babál P Pechánová O Bernátová I Stvrtina S Chronic inhibition of NO synthesis produces myocardial fibrosis and arterial media hyperplasia Histol Histopathol 1997 12 623 9 9225143

[B33] Vrankova S Parohova J Barta A Janega P Simko F Pechanova O Effect of nuclear factor kappa B inhibition on L-NAME-induced hypertension and cardiovascular remodelling J Hypertens 2010 28 Suppl 1 S45 9 10.1097/01.hjh.0000388494.58707.0f 20823716

[B34] Nakmareong S Kukongviriyapan U Pakdeechote P Donpunha W Kukongviriyapan V Kongyingyoes B Sompamit K Phisalaphong C Antioxidant and vascular protective effects of curcumin and tetrahydrocurcumin in rats with L-NAME-induced hypertension Naunyn Schmiedebergs Arch Pharmacol 2011 383 519 29 10.1007/s00210-011-0624-z 21448566

[B35] Spániková A Simoncíková P Ravingerová T Pechánová O Barancík M The effect of chronic nitric oxide synthases inhibition on regulatory proteins in rat hearts Mol Cell Biochem 2008 312 113 20 10.1007/s11010-008-9726-4 18327702

[B36] Kumar S Saravana Kumar M Raja B Efficacy of piperine, an alkaloidal constituent of pepper on nitric oxide, antioxidants and lipid peroxidation markers in L-NAME induced hypertensive rats Int J Res Pharm Sci 2010 1 300 307

[B37] Pechánová O Dobesová Z Cejka J Kunes J Zicha J Vasoactive systems in L-NAME hypertension: the role of inducible nitric oxide synthase J Hypertens 2004 22 167 73 10.1097/00004872-200401000-00026 15106808

[B38] Camacho-Barquero L Villegas I Sánchez-Calvo JM Talero E Sánchez-Fidalgo S Motilva V Alarcón de la Lastra C Curcumin, a Curcuma longa constituent, acts on MAPK p38 pathway modulating COX-2 and iNOS expression in chronic experimental colitis Int Immunopharmacol 2007 7 333 42 10.1016/j.intimp.2006.11.006 17276891

[B39] Farhangkhoee H Khan ZA Chen S Chakrabarti S Differential effects of curcumin on vasoactive factors in the diabetic rat heart Nutr Metab (Lond) 2006 18 3 27 10.1186/1743-7075-3-27 PMC1543622 16848894

[B40] Ramaswami G Chai H Yao Q Lin PH Lumsden AB Chen C Curcumin blocks homocysteine-induced endothelial dysfunction in porcine coronary arteries J Vasc Surg 2004 40 1216 22 10.1016/j.jvs.2004.09.021 15622377

[B41] Haefliger JA Meda P Formenton A Wiesel P Zanchi A Brunner HR Nicod P Hayoz D Aortic connexin43 is decreased during hypertension induced by inhibition of nitric oxide synthase Arterioscler Thromb Vasc Biol 1999 19 1615 22 10.1161/01.ATV.19.7.1615 10397678

[B42] Nadaud S Dupuis M Brocheriou I Haloui M Louedec L Capron F Michel JB Soubrier F Counter-regulation by atorvastatin of gene modulations induced by L-NAME hypertension is associated with vascular protection Vascul Pharmacol 2009 51 253 61 10.1016/j.vph.2009.06.011 19586617

[B43] Guo X Lanir Y Kassab GS Effect of osmolarity on the zero-stress state and mechanical properties of aorta Am J Physiol Heart Circ Physiol 2007 293 H2328 34 10.1152/ajpheart.00402.2007 17573459

[B44] Hu JJ Ambrus A Fossum TW Miller MW Humphrey JD Wilson E Time courses of growth and remodeling of porcine aortic media during hypertension: a quantitative immunohistochemical examination J Histochem Cytochem 2008 56 359 70 18071063 10.1369/jhc.7A7324.2007 PMC2326104

[B45] Hashimoto R Umemoto S Guo F Umeji K Itoh S Kishi H Kobayashi S Matsuzaki M Nifedipine activates PPARgamma and exerts antioxidative actionthrough Cu/ZnSOD independent of blood-pressure lowering in SHRSP J AtherosclerThromb 2010 17 785 95 10.5551/jat.4556 20460829

[B46] Xie L Lin P Xie H Xu C Effects of atorvastatin and losartan onmonocrotaline-induced pulmonary artery remodeling in rats Clin Exp Hypertens 2010 32 547 54 10.3109/10641963.2010.503295 21091363

[B47] Bézie Y Lamazière JM Laurent S Challande P Cunha RS Bonnet J Lacolley P Fibronectin expression and aortic wall elastic modulus in spontaneously hypertensive rats Arterioscler Thromb Vasc Biol 1998 18 1027 34 10.1161/01.ATV.18.7.1027 9672062

[B48] Arribas SM Hinek A González MC Elastic fibres and vascular structure in hypertension Pharmacol Ther 2006 111 771 91 10.1016/j.pharmthera.2005.12.003 16488477

[B49] Joe B Lokesh BR Effect of curcumin and capsaicin on arachidonic acid metabolism and lysosomal enzyme secretion by rat peritoneal macrophages Lipids 1997 32 1173 80 10.1007/s11745-997-0151-8 9397403

[B50] Khalfi F Gressier B Dine T Brunet C Luyckx M Ballester L Cazin M Cazin JC Verapamil inhibits elastase release and superoxide anion production in human neutrophils Biol Pharm Bull 1998 21 109 12 10.1248/bpb.21.109 9514602

[B51] Banerji A Chakrabarti J Mitra A Chatterjee A Effect of curcumin on gelatinase A (MMP-2) activity in B16F10 melanoma cells Cancer Lett 2004 211 235 42 10.1016/j.canlet.2004.02.007 15219947

[B52] Lin CX Rhaleb NE Yang XP Liao TD D'Ambrosio MA Carretero OA Prevention of aortic fibrosis by N-acetyl-seryl-aspartyl-lysyl-proline in angiotensin II-induced hypertension Am J Physiol Heart Circ Physiol 2008 295 H1253 H1261 10.1152/ajpheart.00481.2008 18641275 PMC2544498

[B53] Fitch RM Rutledge JC Wang YX Powers AF Tseng JL Clary T Rubanyi GM Synergistic effect of angiotensin II and nitric oxide synthase inhibitor in increasing aortic stiffness in mice Am J Physiol Heart Circ Physiol 2006 290 H1190 8 16272204 10.1152/ajpheart.00327.2005

[B54] Seccia TM Bettini E Vulpis V Quartaroli M Trist DG Gaviraghi G Pirrelli A Extracellular matrix gene expression in the left ventricular tissue of spontaneously hypertensive rats Blood Press 1999 8 57 64 10.1080/080370599438400 10412884

[B55] Shapiro SD Matrix metalloproteinase degradation of extracellular matrix: biological consequences Curr Opin Cell Biol 1998 10 602 8 10.1016/S0955-0674(98)80035-5 9818170

[B56] Kang HC Nan JX Park PH Kim JY Lee SH Woo SW Zhao YZ Park EJ Sohn DH Curcumin inhibits collagen synthesis and hepatic stellate cell activation in-vivo and in-vitro J Pharm Pharmacol 2002 54 119 26 11829122 10.1211/0022357021771823

[B57] Kocher O Gabbiani G Analysis of alpha-smooth-muscle actin mRNA expression in rat aortic smooth-muscle cells using a specific cDNA probe Differentiation 1987 34 201 9 10.1111/j.1432-0436.1987.tb00067.x 3428507

[B58] Maneen MJ Hannah R Vitullo L DeLance N Cipolla MJ Peroxynitrite diminishes myogenic activity and is associated with decreased vascular smooth muscle F-actin in rat posterior cerebral arteries Stroke 2006 37 894 9 10.1161/01.STR.0000204043.18592.0d 16456123

[B59] Antibodies http://www.antibodies-online.com/clone/1A4/

[B60] Puchtler H Waldrop FS Meloan SN On the mechanism of Mallory's phosphotungstic acid-haematoxylin stain J Microsc 1980 119 383 90 10.1111/j.1365-2818.1980.tb04109.x 6157822

[B61] Liu Y Dolence J Ren J Rao M Sreejayan N Inhibitory effect of dehydrozingerone on vascular smooth muscle cell function J Cardiovasc Pharmacol 2008 52 422 9 10.1097/FJC.0b013e31818aed93 19033821

[B62] Chakravarti N Kadara H Yoon DJ Shay JW Myers JN Lotan D Sonenberg N Lotan R Differential inhibition of protein translation machinery by curcumin in normal, immortalized, and malignant oral epithelial cells Cancer Prev Res(Phila) 2010 3 331 8 10.1158/1940-6207.CAPR-09-0076 PMC2833226 20145189

[B63] Carlier MF Actin: protein structure and filament dynamics J Biol Chem 1991 266 1 4 1985885

